# Supplementary material for: Huangshan Maofeng Green Tea Extracts Prevent Obesity-Associated Metabolic Disorders by Maintaining Homeostasis of Gut Microbiota and Hepatic Lipid Classes in Leptin Receptor Knockout Rats
Source: Foods. 2022 Sep 20;11(19):2939. doi: 10.3390/foods11192939 (PMC9562686; doi:10.3390/foods11192939)
Supplement: Supplementary file 1 [file foods-11-02939-s001.zip › foods-1850659 - supplementary.pdf]

foods MDPI

Supplementary Materials

Table S1. Experimental diet compositions.

Ingredient (g)	Content Percentage
Casein	191
Cornstarch	484
Dextrin	120
Sucrose	66
Soybean oil	24
Lard	19
Cellulose	48
Mineral mix	33
Vitamin mix	10
L-Cystine	3
Line bitartrate	2
TBHQ	0.01
Total	1000
Total energy	
Protein, %	20.6
Fat, %	12.0
Carbohydrate, %	67.4
Energy, Kcal/g	3.616

Table S2. Real-time PCR primers used to analyze gene expression.

Gene	Primer Sequences Forward (5′-3′)	Reverse (5′-3′)
Lxrα	GCTCTGCTCATAGCCATCAG	TGTTGCAGTCTCTCTACTTGGA
Srebf1	TCTCCTGGAGCGAGCATTGA	CAGTGGTGGTAGCCATGCTG
Pparγ	GAAAGACAACAGACAAATCACCAT	CAGCTTCCACGGATCGAAACTG
Thrsp	GCCAGAGTTCACATCCCATC	GGTGCCTTTGATTCCGTGTC
Accα	GGAAGTGGAAGGCACAGTGAAGG	CTGCGGATCTGCTTGAGGACATAG
Fasn	GTTGCTGCTGCTGTGGACCTC	AGGATCACATTGCCGTGGTACTTG
Scd1	TGCTCATGTGCTTCATCCTG	GGGAAACCAGGATGTTCTCC
Hmgcr	CCAAACCCAGTAACCCAAAG	GGTAAAACTGCCAGAGAGAAACACT
18S	GGGTCGGGAGTGGGTAATTT	AGAAACGGCTACCACATCCAA

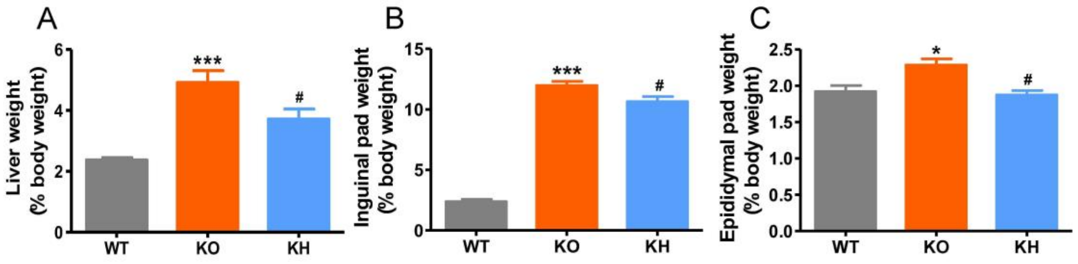

Figure S1. The weights of liver, inguinal fat and epididymal fat relative to whole body weight in *Lepr*$^{-/-}$ rats. Statistic results show liver-to-body weight ratio (**A**); the ratio of inguinal pad weight to body weight (**B**); the ratio of epididymal pad weight to body weight (**C**). Values are the mean ± SEM (n = 6). * $p < 0.05$, *** $p < 0.001$, compared to WT group; # $p < 0.05$ versus KO group.

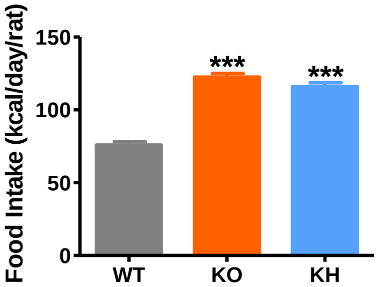

Figure S2. Effects of HTE on food intake in *Lepr*$^{-/-}$ rats. Values are the mean ± SEM (n = 6). *** $p <$ 0.001, compared to WT group.

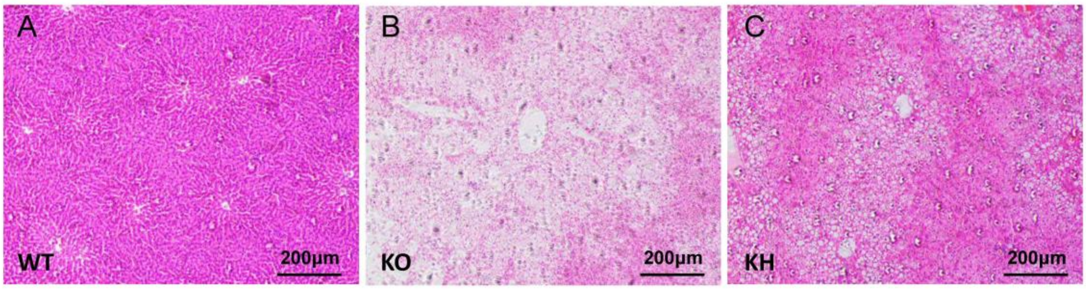

Figure S3. Histopathology image of the liver tissues of *Lepr*$^{-/-}$ rats (H&E, 100×). Representative images of liver H&E staining in WT (**A**), KO (**B**) and KH (**C**) group, respectively.

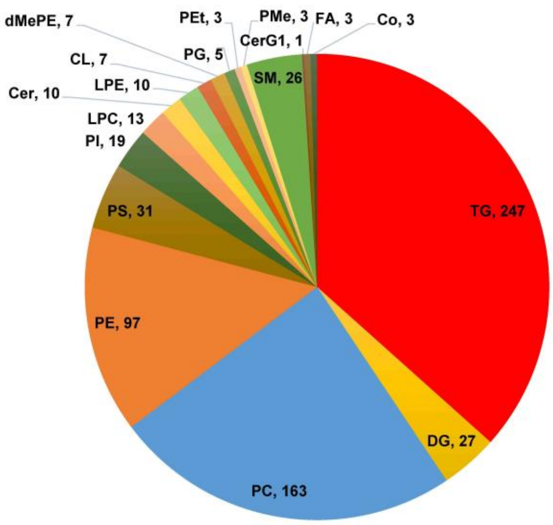

Figure S4. The distribution of lipid classes determined by UHPLC-Orbitrap-MS/MS in liver samples.

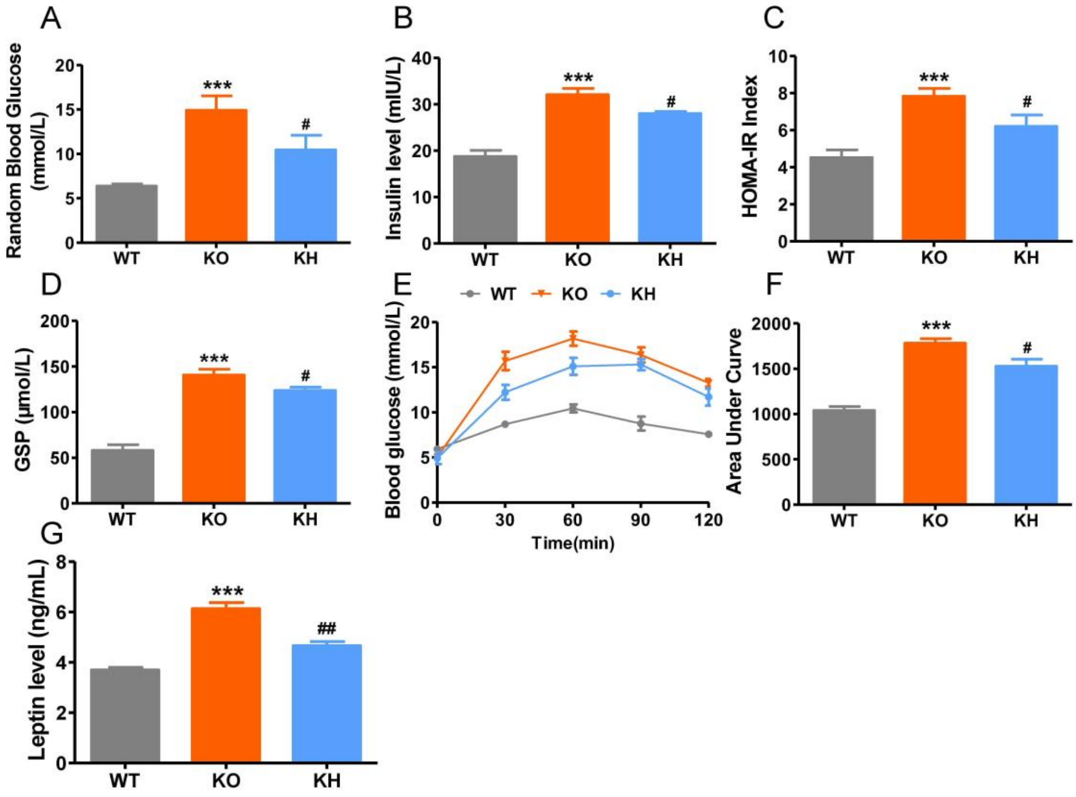

Figure S5. Effects of HTE on glucose tolerance in *Lepr*$^{-/-}$ rats. (**A**) Random blood glucose; (**B**) fasting serum insulin level; (**C**) HOMA-IR; (**D**) glycated serum protein level; (**E**, **F**) oral glucose tolerance test and area under the curve; (**G**) leptin level. Values are the mean ± SEM (n = 4-6). *** p < 0.001, compared to WT group; # p < 0.05, ## p < 0.01, versus KO group.

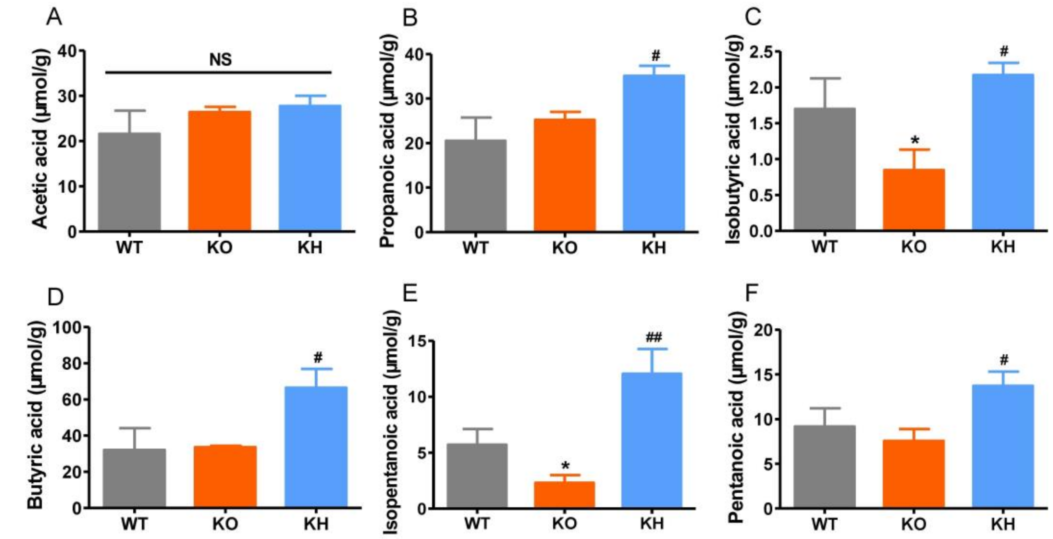

Figure S6. HTE enhanced SCFA production in *Lepr*$^{-/-}$ rats. (**A**) acetic acid level; (**B**) propanoic acid level; (**C**) isobutyric acid level; (**D**) butyric acid level; (**E**) isopentanoic acid level; (**F**) pentanoic acid level. * $p < 0.05$ versus WT group; # p<0.05, ## p<0.01 versus KO group (n = 5, means ± SEM).
